# Adoption and Prescribing Practices of Sodium-Glucose Transport Protein-2 (SGLT-2) Inhibitors and Glucagon-Like Peptide-1 (GLP-1) Agonists in Cardiology: A Cross-Sectional Study in Saudi Arabia

**DOI:** 10.7759/cureus.80610

**Published:** 2025-03-15

**Authors:** Fatema Jamsheer, Abdulaziz Alahmed, Noor Alshamlan, Noor Abdali, Marwah Aljarmal, Najlaa Alsudairy

**Affiliations:** 1 Internal Medicine, Salmaniya Medical Complex, Manama, BHR; 2 General Practice, First Moscow State Medical University, Moscow, RUS; 3 Family Medicine, National Guard Hospital, Jeddah, SAU

**Keywords:** adoption barriers, cardiology practice, cardiometabolic diseases, clinical guidelines, familiarity, glp-1 agonists, heart failure, prescribing practices, safety concerns, sglt2 inhibitors

## Abstract

Background: Heart failure (HF) and cardiometabolic diseases are a significant global health burden, often coexisting in patients, leading to high morbidity and mortality. The management of these conditions is challenging, and new therapeutic approaches are needed. Sodium-glucose transport protein-2 (SGLT-2) inhibitors and glucagon-like peptide-1 (GLP-1)agonists have emerged as promising treatments, demonstrating efficacy in managing both cardiovascular and metabolic outcomes. This study aimed to explore the familiarity, prescribing practices, perceptions, and barriers to the use of SGLT-2 inhibitors and GLP-1 agonists in cardiology practice in Saudi Arabia.

Methods: A cross-sectional, survey-based study was conducted with cardiology professionals across Saudi Arabia. Participants were invited through convenience sampling from academic and community hospitals, private practices, and multi-specialty clinics. The self-administered online survey assessed demographic characteristics, familiarity with SGLT-2 inhibitors and GLP-1 agonists, prescribing practices, perceived efficacy and safety concerns, barriers to adoption, and support for increased use. Descriptive statistics and logistic regression analysis were used to evaluate the data.

Results: A total of 250 cardiology professionals participated, with 46% (n=115) specializing in heart failure, 30.4% (n=76) in general cardiology, 16.4% (n=41) in interventional cardiology, 4.8% (n=12) in electrophysiology, and 2.4% (n=6) in other cardiology specialties. Regarding familiarity with SGLT-2 inhibitors, 28% (n=70) were very familiar, 46.8% (n=117) were somewhat familiar, and 14.4% (n=36) were not familiar at all. For GLP-1 agonists, 25.6% (n=64) were very familiar, 42% (n=105) were somewhat familiar, and 20.8% (n=52) were not familiar. Prescription practices showed that 10.8% (n=27) of participants always prescribed SGLT-2 inhibitors for heart failure, and 8.8% (n=22) always prescribed GLP-1 agonists for cardiometabolic diseases. Factors influencing prescription practices included the patient's clinical condition (57.6%, n=144), clinical guidelines (51.6%, n=129), and recent clinical trial data (39.6%, n=99). The primary barriers to adoption included high cost or insurance issues (40.8%, n=102) and concerns about side effects (36.4%, n=91).

Conclusions: Despite the proven efficacy of SGLT-2 inhibitors and GLP-1 agonists, their adoption in cardiology is limited by familiarity gaps, cost concerns, and safety issues. Enhanced education, better access to guidelines, and addressing cost barriers are crucial steps to improve the adoption of these medications in clinical practice.

## Introduction

The management of heart failure and cardiometabolic diseases represents a major challenge in clinical cardiology. Heart failure, a condition characterized by the heart's inability to pump sufficient blood to meet the body’s needs, is associated with high morbidity, mortality, and healthcare costs worldwide [1]. The prevalence of heart failure is increasing, largely due to an aging population and the rising burden of risk factors such as hypertension, diabetes, and coronary artery disease. Concurrently, cardiometabolic diseases, including diabetes mellitus, obesity, and dyslipidemia, significantly contribute to cardiovascular morbidity and are key risk factors for the development of heart failure. Effective management of these conditions is critical to reducing both the incidence and progression of heart failure, as well as improving the long-term prognosis of patients [2].

In recent years, advances in pharmacotherapy have reshaped the landscape of heart failure and cardiometabolic disease management. The emergence of newer classes of medications, such as sodium-glucose cotransporter-2 inhibitors (SGLT-2) and glucagon-like peptide-1 receptor agonists (GLP-1), has provided new therapeutic options that address both the heart and the metabolism in a way that was previously not possible [3,4]. These medications have demonstrated considerable efficacy not only in managing diabetes and cardiovascular risk factors but also in improving heart failure outcomes, including reducing hospitalization rates, mortality, and disease progression [3,4].

SGLT-2 inhibitors, initially developed as a treatment for type 2 diabetes, have garnered significant attention in recent years due to their beneficial effects on heart failure. These medications work by inhibiting the sodium-glucose cotransporter-2 in the kidneys, which reduces glucose reabsorption and promotes glucosuria. Beyond their glucose-lowering effects, SGLT-2 inhibitors have been shown to have direct benefits in heart failure by reducing blood volume, improving myocardial oxygen supply, and exerting anti-inflammatory and anti-fibrotic effects on the heart [5,6].

GLP-1 receptor agonists are another class of medications that have demonstrated substantial benefits in managing cardiometabolic diseases [7,8]. These medications mimic the actions of endogenous GLP-1, a hormone that enhances insulin secretion, suppresses glucagon release, and slows gastric emptying. In addition to their well-established role in the treatment of type 2 diabetes, GLP-1 agonists have shown promising cardiovascular benefits, including reductions in major adverse cardiovascular events, weight loss, and improvements in lipid profiles.

Despite the clear clinical benefits of SGLT-2 inhibitors and GLP-1 agonists [7,8], their adoption in routine clinical practice has been slow. Several factors contribute to this lag in adoption, including limited familiarity with these medications among clinicians, particularly those who are not involved in diabetes management, concerns about the safety profiles of these medications, and financial barriers related to their high cost and insurance coverage [9,10].

The adoption of these medications in cardiology practice is particularly important, as heart failure and cardiometabolic diseases are often comorbid conditions. However, cardiologists may face challenges in keeping up with rapidly evolving pharmacological treatments, particularly when medications originally developed for diabetes are now being used in heart failure.

Given the growing body of evidence supporting the efficacy of SGLT-2 inhibitors and GLP-1 agonists, it is essential to understand how these medications are being adopted by cardiology practitioners and what barriers exist to their use. This study aimed to assess the familiarity, prescribing practices, perceptions, and barriers related to the use of SGLT-2 inhibitors and GLP-1 agonists in cardiology practice in Saudi Arabia. By identifying these factors, the study seeks to inform strategies to promote the wider adoption of these medications in the management of heart failure and cardiometabolic diseases.

## Materials and methods

Study design and participants

This was a cross-sectional, survey-based study aimed at evaluating the adoption, prescribing practices, and perceptions of SGLT-2 inhibitors and GLP-1 agonists in cardiology practice for heart failure and cardiometabolic protection. Cardiology professionals were invited to participate, including general cardiologists, heart failure specialists, interventional cardiologists, electrophysiologists, and other cardiology specialists. Participants were selected through a convenience sampling approach from academic and community hospitals, private practices, and multi-specialty clinics across Saudi Arabia. The study was approved by the Institutional Review Board of the Ministry of Health (HAPO-10-Z-001), Kingdom of Saudi Arabia with approval number REC-2024-824.

Survey instrument and data collection

The survey instrument was a self-administered, anonymous online questionnaire designed to assess respondents' familiarity with, prescribing practices for, and perceptions of SGLT-2 inhibitors and GLP-1 agonists. The questionnaire was developed based on a comprehensive literature review and consultations with experts in cardiology and pharmacology. It was divided into sections that focused on demographic information, familiarity with the medications, prescribing practices, perceived efficacy and safety concerns, barriers to adoption, and support for increasing medication adoption.

The survey was distributed electronically to cardiologists across Saudi Arabia from January 2025 to February 2025. Invitations were sent via email and professional networks, followed by a reminder after two weeks. Participation was voluntary, and all responses were kept confidential. No compensation was provided for participation.

Variables and outcome measures

The study aimed to assess several key variables related to the adoption of SGLT-2 inhibitors and GLP-1 agonists. Demographic variables included the specialty of the respondent, years of practice, and the type of hospital or clinic in which the participant was employed. The familiarity with SGLT-2 inhibitors and GLP-1 agonists was assessed by asking respondents to report their self-reported level of familiarity, categorized as "very familiar," "somewhat familiar," or "not familiar at all."

Prescription practices were evaluated by asking respondents how often they prescribed SGLT-2 inhibitors for heart failure and GLP-1 agonists for cardiometabolic diseases. Response categories ranged from "always," "frequently," and "occasionally," to "never." Factors influencing prescription practices were also explored, with respondents asked to identify factors such as patient clinical condition, clinical guidelines, and recent clinical trial data.

The perceived efficacy of the medications was assessed by asking respondents to rate the effectiveness of SGLT-2 inhibitors for heart failure and GLP-1 agonists for cardiometabolic diseases, using categories of "highly effective," "moderately effective," or "not effective." Safety concerns associated with these medications were also addressed, with respondents asked about their concerns regarding side effects such as renal dysfunction, diabetic ketoacidosis, and gastrointestinal issues.

Barriers to adoption of these medications were explored through questions about cost, insurance coverage, side effects, and other factors that might influence the decision to prescribe these medications. Additionally, the survey assessed the level of awareness of clinical guidelines related to the use of SGLT-2 inhibitors and GLP-1 agonists and how often these guidelines were utilized in prescribing practices.

Finally, the survey assessed the support for increasing the adoption of these medications. Respondents were asked about the need for further education and training, improved patient education materials, increased evidence from clinical trials, and better insurance coverage to support the wider use of SGLT-2 inhibitors and GLP-1 agonists.

Questionnaire development and validation

The questionnaire was developed based on a review of the literature and expert opinions. The initial version of the questionnaire included questions related to demographic information, familiarity with SGLT-2 inhibitors and GLP-1 agonists, prescribing practices, perceived barriers to adoption, and factors influencing prescription decisions.

To ensure content validity, the questionnaire was reviewed by a panel of five cardiology experts, including physicians with expertise in heart failure, cardiology, and pharmacology. They provided feedback on the clarity, relevance, and completeness of the questions. After incorporating their suggestions, a pre-test was conducted with 30 cardiology professionals to evaluate the clarity and understanding of the questions (Appendix A). Based on feedback from the pre-test, minor revisions were made to improve question wording and response options.

Statistical analysis

Descriptive statistics were used to summarize the demographic characteristics of the participants, as well as their responses to the survey questions. Continuous variables were summarized using means and standard deviations, while categorical variables were reported as frequencies and percentages. To compare the prescribing practices of different cardiology specialties, chi-square tests were performed. A p-value of less than 0.05 was considered statistically significant.

To investigate the factors influencing the prescribing practices for SGLT-2 inhibitors and GLP-1 agonists, logistic regression analysis was conducted. The independent variables included familiarity with the medications, awareness of clinical guidelines, the clinical condition of the patients, and insurance coverage. Odds ratios (OR) with 95% confidence intervals (CI) were calculated to assess the strength of association between these factors and the likelihood of prescribing the medications. All statistical analyses were performed using SPSS software version 27.0 (IBM Corp., Armonk, NY).

## Results

Demographics and practice information

A total of 250 cardiology professionals participated in this survey. The specialties represented were general cardiology (n=76), heart failure (n=115), interventional cardiology (n=41), electrophysiology (n=12), and other cardiology specialties (n=6). Regarding years of practice, 15.6% (n=39) of respondents had less than 5 years of experience, 34.8% (n=87) had 5-10 years, 28.4% (n=71) had 11-20 years, and 21.2% (n=53) had more than 20 years. The majority of respondents worked in academic hospitals (n=99, 39.6%), followed by community hospitals (n=69, 27.6%), private practices (n=59, 23.6%), and multi-specialty clinics (n=21, 8.4%) (Table 1).

**Table 1:** Demographics and practice information (N=250). Data presented as n (%) of total respondents (N=250).

Variable	Response	Frequency (%)
Specialization	General cardiology	76 (30.4%)
	Heart failure	115 (46%)
	Interventional cardiology	41 (16.4%)
	Electrophysiology	12 (4.8%)
	Other (e.g., academic cardiology)	6 (2.4%)
Years of practice	Less than 5 years	39 (15.6%)
	5-10 years	87 (34.8%)
	11-20 years	71 (28.4%)
	More than 20 years	53 (21.2%)
Work setting	Academic hospital	99 (39.6%)
	Private practice	59 (23.6%)
	Community hospital	69 (27.6%)
	Multi-specialty clinic	21 (8.4%)
	Other	2 (0.8%)

Familiarity with SGLT-2 inhibitors and GLP-1 agonists

When asked about familiarity with SGLT-2 inhibitors, 28% (n=70) of respondents were very familiar, 46.8% (n=117) were somewhat familiar, and 14.4% (n=36) were not familiar at all. A smaller group (10.8%, n=27) was aware of the drug but did not use it. In terms of GLP-1 agonists, 25.6% (n=64) were very familiar, 42% (n=105) were somewhat familiar, and 20.8% (n=52) were not familiar. Only 11.6% (n=29) were aware of the drug but did not prescribe it (Table 2).

**Table 2:** Knowledge and familiarity with SGLT2 inhibitors and GLP-1 agonists (N=250) SGLT-2: sodium-glucose cotransporter-2; GLP-1: glucagon-like peptide-1. Data presented as n (%) of total respondents (N=250).

Variable	Response	Frequency (%)
Familiarity with SGLT-2 inhibitors	Very familiar	70 (28%)
	Somewhat familiar	117 (46.8%)
	Not familiar at all	36 (14.4%)
	Aware but do not use	27 (10.8%)
Familiarity with GLP-1 agonists	Very familiar	64 (25.6%)
	Somewhat familiar	105 (42%)
	Not familiar at all	52 (20.8%)
	Aware but do not use	29 (11.6%)

Prescription practices and influencing factors

Regarding prescription practices for heart failure, 10.8% (n=27) of respondents always prescribed SGLT-2 inhibitors, 38.8% (n=97) prescribed them frequently, 32.4% (n=81) occasionally, and 18% (n=45) never prescribed them. For GLP-1 agonists, 8.8% (n=22) always prescribed them for cardiometabolic diseases, 34.8% (n=87) prescribed them frequently, 35.2% (n=88) occasionally, and 21.2% (n=53) never prescribed them. The most common factors influencing prescription were patient clinical condition (57.6%, n=144), clinical guidelines (51.6%, n=129), and recent clinical trial data (39.6%, n=99) (Table 3).

**Table 3:** Prescription practices and influencing factors for SGLT2 inhibitors and GLP-1 agonists (N=250) SGLT-2: sodium-glucose cotransporter-2; GLP-1: glucagon-like peptide-1. Data presented as n (%) of total respondents (N=250).

Variable	Response	Frequency (%)
Frequency of prescribing SGLT2 inhibitors for heart failure	Always	27 (10.8%)
	Frequently	97 (38.8%)
	Occasionally	81 (32.4%)
	Never	45 (18%)
Frequency of prescribing GLP-1 agonists for cardiometabolic diseases	Always	22 (8.8%)
	Frequently	87 (34.8%)
	Occasionally	88 (35.2%)
	Never	53 (21.2%)
Factors influencing prescription	Patient’s clinical condition	144 (57.6%)
	Recent clinical trial data	99 (39.6%)
	Clinical guidelines	129 (51.6%)
	Patient's cost concerns	74 (29.6%)
	Physician experience	112 (44.8%)

Perceived efficacy and safety concerns

When asked about the perceived efficacy of SGLT-2 inhibitors in heart failure, 32.8% (n=82) of respondents considered them highly effective, 45.2% (n=113) moderately effective, and 13.6% (n=34) considered them not effective. Regarding GLP-1 agonists, 29.2% (n=73) of respondents viewed them as highly effective for cardiometabolic outcomes, 44.4% (n=111) as moderately effective, and 9.6% (n=24) as not effective. The most common safety concerns for SGLT-2 inhibitors were the risk of renal dysfunction (50.4%, n=126), diabetic ketoacidosis (37.6%, n=94), and urinary tract infections (28%, n=70). For GLP-1 agonists, gastrointestinal side effects (34.8%, n=87) and the risk of pancreatitis (22.4%, n=56) were the most common concerns (Table 4).

**Table 4:** Perceived efficacy and safety concerns of SGLT2 inhibitors and GLP-1 agonists (N=250) SGLT-2, sodium-glucose cotransporter-2; GLP-1, glucagon-like peptide-1. Data presented as n (%) of total respondents (N=250).

Variable	Response	Frequency (%)
Perceived efficacy of SGLT2 inhibitors in heart failure	Highly effective	82 (32.8%)
	Moderately effective	113 (45.2%)
	Not effective	34 (13.6%)
	Unsure	21 (8.4%)
Perceived efficacy of GLP-1 agonists for cardiometabolic outcomes	Highly effective	73 (29.2%)
	Moderately effective	111 (44.4%)
	Not effective	24 (9.6%)
	Unsure	42 (16.8%)
Concerns about the safety of SGLT2 inhibitors	Risk of renal dysfunction	126 (50.4%)
	Risk of hypoglycemia	73 (29.2%)
	Risk of diabetic ketoacidosis	94 (37.6%)
	Risk of urinary tract infections	70 (28%)
	No concerns	48 (19.2%)
Concerns about the safety of GLP-1 agonists	Gastrointestinal side effects	87 (34.8%)
	Risk of pancreatitis	56 (22.4%)
	Risk of weight loss	61 (24.4%)
	No concerns	87 (34.8%)

Barriers to adoption and guidelines utilization

The primary barriers to adopting SGLT-2 inhibitors were high cost or insurance issues (40.8%, n=102) and concerns about side effects (36.4%, n=91). For GLP-1 agonists, the barriers were similar, with 41.6% (n=104) citing high cost and 39.6% (n=99) concerned about side effects. Regarding clinical guidelines utilization, 44% (n=110) of respondents were very aware of the guidelines, 38.4% (n=96) were somewhat aware, and 16% (n=40) were not very aware. Only 1.6% (n=4) were not aware of the guidelines at all. In total, 33.6% (n=84) of respondents reported being fully aware of the current guidelines, while 42.4% (n=106) were somewhat aware (Table 5).

**Table 5:** Barriers to adoption and utilization of guidelines for SGLT-2 inhibitors and GLP-1 agonists (N=250) SGLT-2, sodium-glucose cotransporter-2; GLP-1, glucagon-like peptide-1. Data presented as n (%) of total respondents (N=250).

Variable	Response	Frequency (%)
Barriers to adopting SGLT-2 inhibitors	Lack of familiarity with the drug	34 (13.6%)
	Concerns about side effects	91 (36.4%)
	High cost or insurance issues	102 (40.8%)
	Insufficient clinical evidence	56 (22.4%)
	Other (Please specify)	27 (10.8%)
Barriers to adopting GLP-1 agonists	Lack of familiarity with the drug	49 (19.6%)
	Concerns about side effects	99 (39.6%)
	High cost or insurance issues	104 (41.6%)
Guidelines utilization for prescription decisions	Very much	110 (44%)
	Somewhat	96 (38.4%)
	Not much	40 (16%)
	Not at all	4 (1.6%)
Awareness of current guidelines	Fully aware	84 (33.6%)
	Somewhat aware	106 (42.4%)
	Not aware	36 (14.4%)
	Not applicable	24 (9.6%)

Support for increasing adoption and associations

To increase the adoption of SGLT-2 inhibitors and GLP-1 agonists, 52.8% (n=132) of respondents supported more clinical education and training, 45.6% (n=114) supported improved patient education materials, 39.2% (n=98) wanted more evidence from clinical trials, and 49.2% (n=123) advocated for better insurance coverage. When asked about the impact of increased adoption on outcomes, 36.8% (n=92) believed it would significantly improve patient outcomes, while 44.4% (n=111) thought it would somewhat improve outcomes, and 12.4% (n=31) believed it would have no significant impact (Table 6).

**Table 6:** Support for adoption and perceived impact on outcomes (N=250) SGLT-2, sodium-glucose cotransporter-2; GLP-1, glucagon-like peptide-1. Data presented as n (%) of total respondents (N=250).

Variable	Response	Frequency (%)
Additional support for increasing adoption	More clinical education and training	132 (52.8%)
	Improved patient education materials	114 (45.6%)
	Increased evidence from clinical trials	98 (39.2%)
	Better insurance coverage and affordability	123 (49.2%)
Increased adoption improves outcomes	Yes, significantly	92 (36.8%)
	Yes, somewhat	111 (44.4%)
	No, not significantly	31 (12.4%)
	Unsure	16 (6.4%)

Comparison of prescribing practices across specializations

Prescribing practices for SGLT2 inhibitors and GLP-1 agonists differed significantly across specialties. General cardiologists were more likely to prescribe SGLT-2 inhibitors (46.1%, n=35) and GLP-1 agonists (29%, n=22) compared to other specialties (p=0.003). Heart failure specialists prescribed both medications at higher rates, with 41.7% (n=48) prescribing SGLT2 inhibitors and 43.5% (n=50) prescribing GLP-1 agonists. In contrast, interventional cardiologists had lower prescription rates for both drug classes (SGLT-2 inhibitors: 29.3%, n=12; GLP-1 agonists: 17.1%, n=7), and electrophysiologists showed similar trends (SGLT2 inhibitors: 33.3%, n=4; GLP-1 agonists: 25%, n=3) (Table 7).

**Table 7:** Comparison of prescribing practices for SGLT2 inhibitors and GLP-1 agonists across different specializations (N=250) SGLT-2: sodium-glucose cotransporter-2; GLP-1: glucagon-like peptide-1. Data presented as n (%) of respondents from each specialization. p-values were calculated using the Chi-square test.

Specialization	Prescribe SGLT2 inhibitors (%)	Prescribe GLP-1 agonists (%)	p-value	Chi-square value
General cardiology	35 (46.1%)	22 (29%)	0.003	8.858
Heart failure	48 (41.7%)	50 (43.5%)	0.087	2.875
Interventional cardiology	12 (29.3%)	7 (17.1%)	0.001	10.929
Electrophysiology	4 (33.3%)	3 (25%)	0.421	0.643
Other	1 (16.7%)	2 (33.3%)	0.263	1.281

Regression analysis of factors influencing prescription of SGLT-2 inhibitors and GLP-1 agonists

Logistic regression analysis indicated that familiarity with the drug (SGLT-2 inhibitors OR=1.25, 95% CI=1.05-1.56, p=0.002; GLP-1 agonists OR=1.42, 95% CI=1.13-1.74, p 0.002), awareness of clinical guidelines (SGLT-2 inhibitors OR=1.32, 95% CI=1.11-1.56, p=0.005; GLP-1 agonists OR=1.35, 95% CI=1.15-1.56, p=0.005), and patient clinical condition (SGLT-2 inhibitors OR=1.41, 95% CI=1.15-1.71, p=0.023; GLP-1 agonists OR=1.28, 95% CI=1.05-1.53, p=0.023) were significant predictors for prescribing both SGLT-2 inhibitors and GLP-1 agonists. Additionally, insurance coverage was a significant factor for GLP-1 agonist prescribing (OR=1.21, 95% CI=1.01-1.42, p=0.037) (Table 8).

**Table 8:** Logistic regression analysis of factors influencing prescription of SGLT-2 inhibitors and GLP-1 agonists (N=250). Odds ratios (OR) with 95% confidence intervals (CI) are presented for each variable in relation to the prescription of SGLT-2 inhibitors and GLP-1 agonists. p-values indicate the statistical significance of differences between the two drugs for each variable.

Variable	SGLT2 inhibitors (OR (95% CI))	GLP-1 agonists (OR (95% CI))	p-value
Familiarity with drug	1.25 (1.05–1.56)	1.42 (1.13–1.74)	0.002
Years of Practice	1.04 (0.95–1.16)	1.06 (0.96–1.17)	0.210
Clinical guidelines awareness	1.32 (1.11–1.56)	1.35 (1.15–1.56)	0.005
Patient's clinical condition	1.41 (1.15–1.71)	1.28 (1.05–1.53)	0.023
Insurance coverage	1.26 (1.05–1.51)	1.21 (1.01–1.42)	0.037

## Discussion

This survey-based study aimed to assess the adoption, prescribing practices, and perceptions of SGLT-2 inhibitors and GLP-1 agonists in cardiology practice for heart failure and cardiometabolic protection in Saudi Arabia. The results highlight several key trends and areas of interest regarding familiarity with these medications, their prescription in clinical practice, perceived efficacy, safety concerns, and the barriers to their adoption. The findings underscore the growing recognition of the importance of these medications in cardiology while also revealing challenges that may impede their broader adoption.

Our study revealed that while cardiology professionals exhibited varying degrees of familiarity with both SGLT-2 inhibitors and GLP-1 agonists, a significant portion of respondents reported being only somewhat familiar or unfamiliar with these medications. Notably, 46.8% of cardiologists were "somewhat familiar" with SGLT-2 inhibitors and 42% with GLP-1 agonists, while fewer reported being "very familiar." These findings are consistent with prior research indicating that despite the increasing evidence supporting the efficacy of these medications in heart failure and cardiometabolic disease, their adoption has been slow, particularly among specialists who may not be directly involved in prescribing them [5,11]. This is reflective of a broader trend in cardiology, where there remains a lag in the integration of newer pharmacological therapies into practice, possibly due to insufficient exposure or the need for more robust clinical education.

The study demonstrated that prescription rates for both SGLT-2 inhibitors and GLP-1 agonists varied widely, with general cardiologists and heart failure specialists showing higher rates of prescribing these drugs compared to other subspecialties, such as interventional cardiology and electrophysiology. This discrepancy may reflect the direct relevance of these medications to the treatment of heart failure and cardiometabolic diseases, conditions more commonly managed by general cardiologists and heart failure specialists. The lower prescription rates among interventional cardiologists and electrophysiologists could be attributed to the more procedural nature of these specialties, which often focus on mechanical interventions rather than pharmacological management of chronic conditions.

Several factors influenced prescribing practices, with clinical condition, awareness of clinical guidelines, and recent clinical trial data being the most commonly cited reasons for prescribing these medications. These findings are in line with current literature that emphasizes the role of evidence-based guidelines in shaping clinical decisions [12-14]. However, despite the availability of robust data on the efficacy of SGLT-2 inhibitors and GLP-1 agonists, our study found that only 44% of cardiologists reported being "very aware" of clinical guidelines for their use. This highlights a critical area for improvement in clinical education and guideline dissemination.

Respondents generally considered both SGLT-2 inhibitors and GLP-1 agonists to be moderately to highly effective in treating heart failure and managing cardiometabolic outcomes, respectively. Notably, 78% of respondents viewed SGLT-2 inhibitors as moderately or highly effective for heart failure, while 73.6% considered GLP-1 agonists effective for cardiometabolic conditions. These perceptions are consistent with findings from recent clinical trials that have demonstrated the beneficial effects of these drugs on heart failure outcomes and cardiovascular risk factors.

Barriers to the adoption of SGLT-2 inhibitors and GLP-1 agonists were primarily related to cost and concerns about side effects. High costs and issues related to insurance coverage were the most commonly cited barriers, which mirrors the challenges faced globally in terms of access to newer medications. In particular, the high cost of GLP-1 agonists can be prohibitive, especially in healthcare systems with limited coverage for these drugs. Furthermore, while clinical trial data continue to support the effectiveness of these medications, the perceived safety risks and potential side effects likely contribute to hesitancy in prescribing these agents, particularly in patients with multiple comorbidities [15].

Interestingly, guideline utilization appeared to be a moderating factor in prescribing decisions. Respondents who reported being more familiar with clinical guidelines for the use of these medications were more likely to prescribe them [15,16]. This suggests that improving the dissemination and awareness of these guidelines could be a critical step in enhancing the adoption of SGLT-2 inhibitors and GLP-1 agonists in clinical practice.

Limitations

Several limitations should be considered when interpreting the results of this study. First, the use of a convenience sampling method may limit the generalizability of the findings to the broader population of cardiologists in Saudi Arabia. Additionally, the self-reported nature of the survey responses may introduce response biases, as participants may overestimate their familiarity with the medications or the frequency with which they prescribe them. Furthermore, while the study focused on cardiologists, it did not assess the role of other healthcare providers, such as primary care physicians, who may also play a significant role in the adoption of these therapies.

## Conclusions

In conclusion, this study highlights the growing recognition of the importance of SGLT-2 inhibitors and GLP-1 agonists in cardiology but also reveals significant gaps in familiarity, prescribing practices, and guideline utilization. While these medications show promise in the management of heart failure and cardiometabolic diseases, further education, dissemination of clinical guidelines, and addressing barriers such as cost and safety concerns are essential to increasing their adoption in clinical practice. Targeted interventions, including professional education and improved patient access, could facilitate the widespread use of these medications, ultimately improving patient outcomes in cardiology.

## Appendices

Appendix A

**Table 9:** Survey questionnaire.

Demographics						
1. Age	☐ 25-34	☐ 35-44	☐ 45-54	☐ 55+		
2. Gender	☐ Male	☐ Female	☐ Prefer not to say			
3. Specialization	☐ Cardiology	☐Internal Medicine	☐ Endocrinology	☐ Other		
4. Years of Experience	☐0-5	☐6-10	☐ 11-20	☐ 21+		
Familiarity and prescription patterns						
5. Familiarity with SGLT2 inhibitors/GLP-1 agonists	☐ Very familiar	☐ Somewhat familiar	☐ Not familiar			
6. Ever prescribed for heart failure or cardiometabolic diseases?	☐ Yes, regularly	☐ Yes, occasionally	☐ No			
7. Prescribing frequency (SGLT2 inhibitors for heart failure)	☐ Always	☐ Often	☐ Rarely	☐ Never		
8. Prescribing frequency (GLP-1 agonists for cardiometabolic diseases)	☐ Always	☐ Often	☐ Rarely	☐ Never		
Barriers to adoption						
9. Main barriers to prescribing SGLT2 inhibitors and GLP-1 agonists: (Check all that apply)	☐ Lack of evidence	☐ High cost	☐ Patient factors	☐ Lack of familiarity	☐ Availability of alternatives	☐ Other
10. Need for more education/training to improve adoption?	☐ Yes	☐ No	☐ Maybe			
Perceptions of efficacy						
11. Effectiveness of SGLT2 inhibitors for heart failure	☐ Very effective	☐ Effective	☐ Neutral	☐ Not effective		
12. Effectiveness of GLP-1 agonists for cardiometabolic diseases	☐ Very effective	☐ Effective	☐ Neutral	☐ Not effective		
